# Retropharyngeal Emphysema Following Local Palate Trauma

**DOI:** 10.7759/cureus.32029

**Published:** 2022-11-29

**Authors:** Abdulaziz S AlEnazi, Zahraa Jumah A AlMuhanna, Aqeelah S Alfaraj, Hussain Abduljabbar A AlKhawaja, Sarah K AlTamimi, Abdulmalik S Alsaied, Mona MohammedSaleh Ashoor

**Affiliations:** 1 Otolaryngology - Head and Neck Surgery, Imam Abdulrahman bin Faisal University, King Fahad University Hospital, Khobar, SAU; 2 College of Medicine, Al-Baha University, Al-Baha, SAU; 3 College of Medicine, Imam Abdulrahman bin Faisal University, Dammam, SAU; 4 Radiology, King Fahad University Hospital, Khobar, SAU; 5 Radiology, Dammam Medical Complex, Dammam, SAU; 6 Otolaryngology, King Fahad University Hospital, Khobar, SAU; 7 Otolaryngology - Head and Neck Surgery, Imam Abdulrahman Bin Faisal University, King Fahad University Hospital, Khobar, SAU

**Keywords:** emphysema, palate trauma, retropharyngeal emphysema, oropharyngeal trauma, retropharyngeal space

## Abstract

Retropharyngeal emphysema (RPE) is a condition that occurs when air is trapped in the retropharyngeal space. It is a rare condition that is either spontaneous or secondary to various etiologies. A case of a three-year-old patient with retropharyngeal emphysema secondary to local palate trauma was presented to King Fahd Hospital of the University. The patient was further investigated by flexible nasopharyngoscopy; however, it showed no additional complications. The patient was admitted to the hospital and managed conservatively with analgesia and antibiotics. Lateral neck X-ray showed complete resolution of retropharyngeal emphysema a few days after admission. The patient was discharged on oral antibiotics and a follow-up after one week was arranged. Upon follow-up, the patient’s condition improved with no further complications.

## Introduction

Subcutaneous emphysema (SE) is a condition that occurs when air is trapped in the subcutaneous layer of the skin. It is a well-documented condition. However, retropharyngeal emphysema (RPE), where the air occupies the retropharyngeal space, is a rare condition that can result from various etiologies, including spontaneous, iatrogenic, or traumatic origin. It is crucial to ensure airway patency and investigate the patient to rule out other complications, including pneumomediastinum, pneumothorax, or pneumopericardium [1,2].

Herein, we report a case of retropharyngeal emphysema proceeded by local trauma to the soft palate with a comprehensive literature review, including the etiology, associated findings, and management.

## Case presentation

A three-year-old male patient with no significant medical history presented to the emergency department of King Fahad Hospital with a history of oral trauma five hours prior to his presentation. The parents stated a history of the patient playing with a plastic toy, which had slightly sharp edges, and putting it inside his mouth. His sibling accidentally pushed it further inside. The patient began to have minimal bleeding, blood-tinged saliva, and severe oral pain. The bleeding stopped shortly after; however, he was continuously crying along with refusing to be fed.

**Figure 1:**
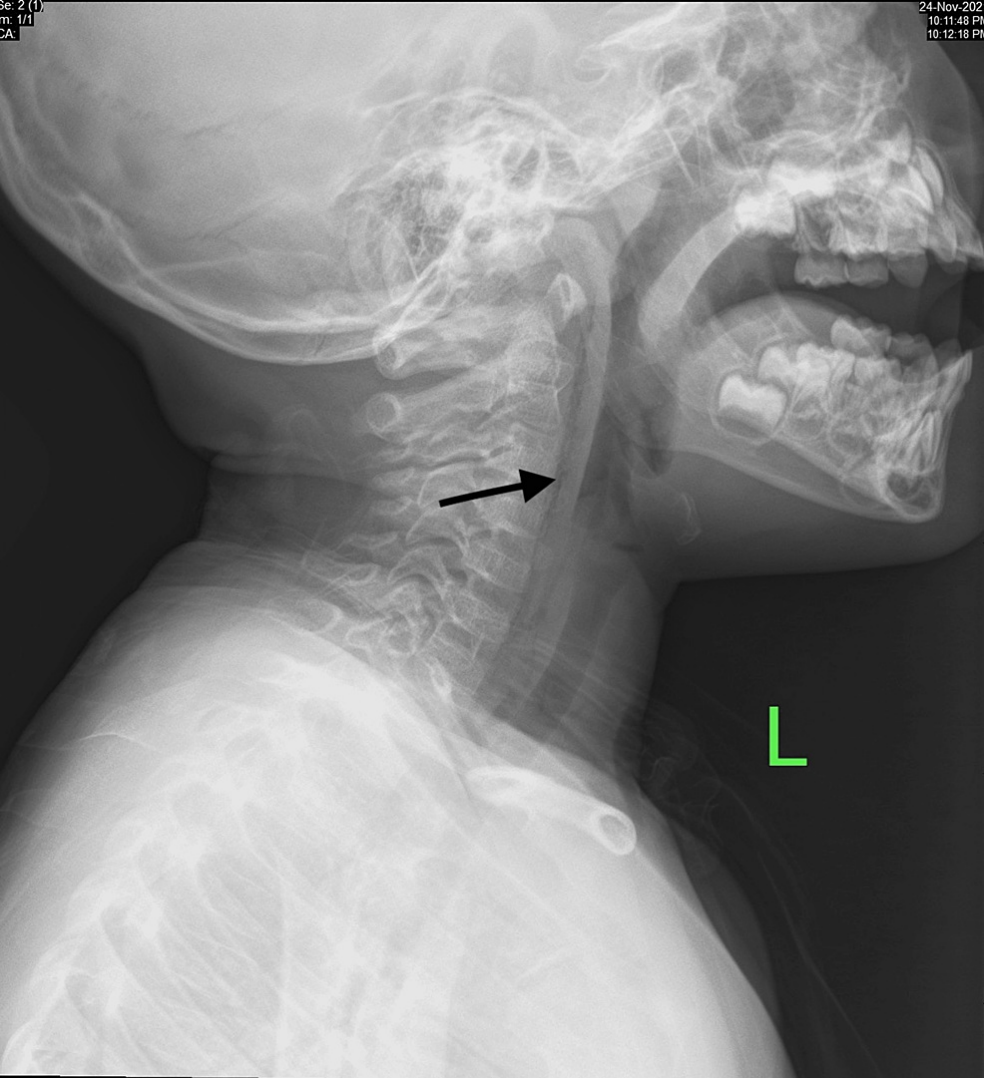
Lateral soft tissue radiography of the neck demonstrates a linear radiolucent line representing air in the retropharyngeal space

Vital signs upon presentation were within normal range. On examination, there was a linear superficial laceration over the soft palate and a small abrasion with sloughed tissue over the posterior pharyngeal wall with no active bleeding or obvious penetration. Flexible nasopharyngoscopy revealed sloughed tissue over the left posterior pharyngeal wall and bilateral mobile vocal folds with no bulging or edema. Lateral soft tissue radiography of the neck demonstrated a linear air column in the retropharyngeal space (Figure 1), whereas chest radiography was unremarkable.

**Figure 2:**
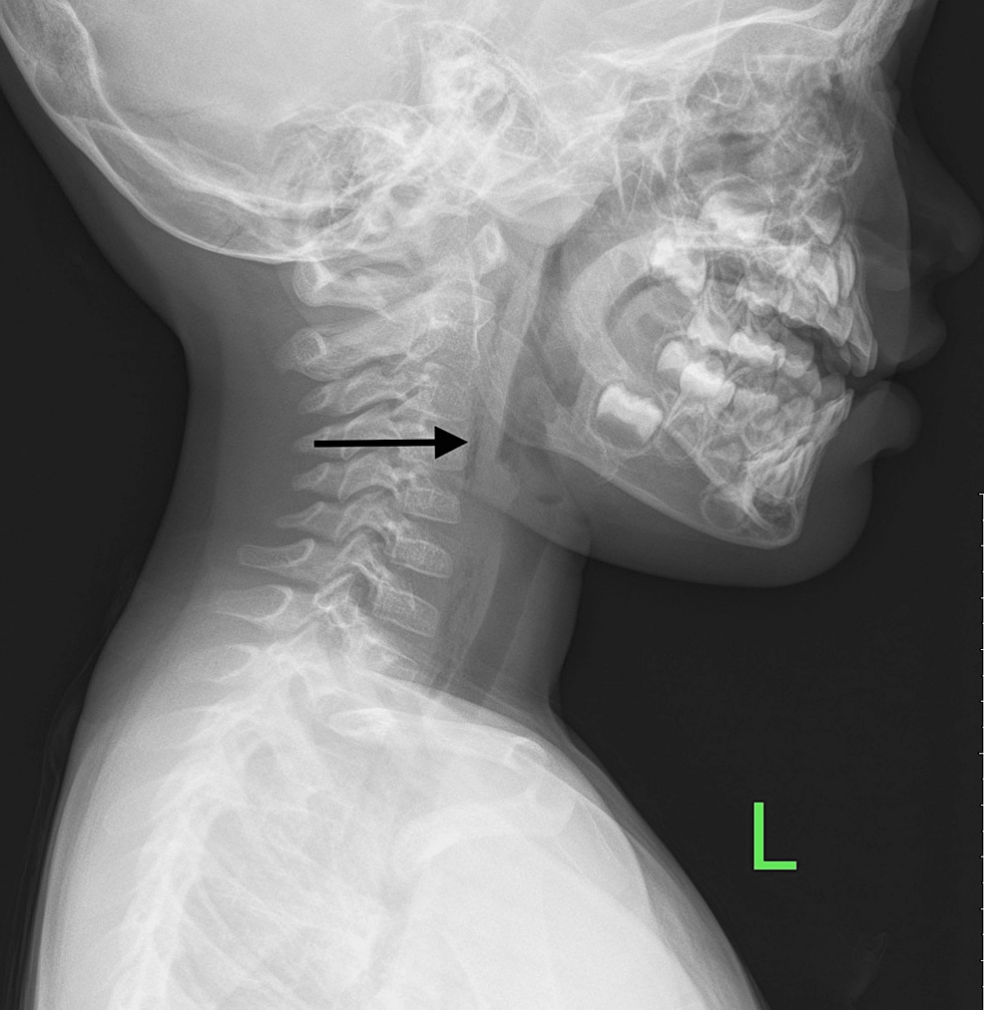
Day two: lateral soft tissue radiography of the neck showed improvement, however, there was incomplete resolution of the retropharyngeal air

The patient was admitted to the hospital and managed conservatively with analgesia and intravenous prophylactic antibiotic for three days. A series of lateral soft tissue radiographs of the neck were obtained during his hospital stay (Figure 2).

**Figure 3:**
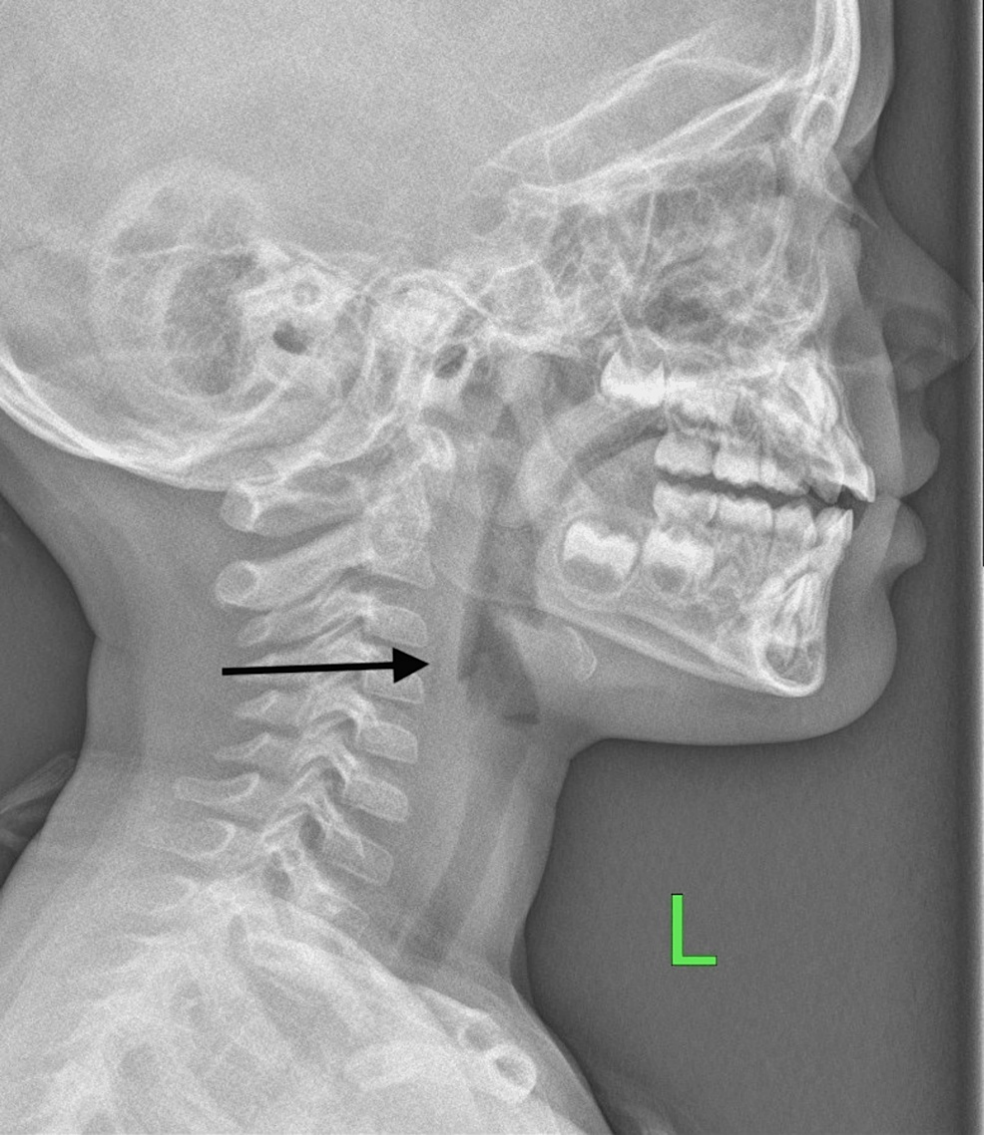
Day three: lateral soft tissue radiography of the neck showed complete resolution of the retropharyngeal air

On the third day, the lateral neck x-ray showed complete resolution of retropharyngeal emphysema (Figure 3). Subsequently, the patient was discharged home on oral antibiotics and was arranged for an outpatient clinic appointment after one week. Upon follow-up, he did not have any active complaints, the patient was improving, and ultimately recovered; therefore, no further follow-up was arranged.

## Discussion

The retropharyngeal space (RPS) is a deep compartment of the neck located between the middle (pretracheal) layer and the deep (prevertebral) layer of the deep cervical fascia. It is a potential space bounded anteriorly by the buccopharyngeal fascia, posteriorly by the prevertebral fascia, and laterally by the carotid sheath. It starts at the base of the skull and terminates at the mediastinum at the level of the thoracic vertebra (T1-T6). The specific anatomic termination of RPS in relation to the thoracic vertebrae varies depending on where the alar fascia fuses with the visceral fascia, its significance comes from its proximity to vital structures - of note are the carotid sheath and mediastinum [3,4]. The retropharyngeal space may host several diseases, thus, differential diagnosis of any RPS disease should include inflammatory reactions, lymph node metastasis, and rarely, primary tumors [5,6]

The etymology of the word emphysema is derived from the word “emphusan” in Latin, which means “to inflate,” the literal meaning of emphysema is to be distended by air or other gases [7,8]. Retropharyngeal emphysema (RPE) is a rare, reported clinical entity. It is the result of free air spreading through the loose connective tissues dissecting the fascial planes of the neck [2].

When air is trapped in the retropharyngeal space, serious complications might develop, air can penetrate through the alar fascia and accumulate in the dangerous space, the so-called Grodinsky and Holyoke’s space. Further serious complications of retropharyngeal emphysema are pneumomediastinum, pneumothorax, and pneumopericardium [9].

Retropharyngeal emphysema can be primary or secondary as a result of various etiologies. Although rare, primary spontaneous retropharyngeal emphysema has been reported multiple times, the diagnosis is only made after other etiologies are excluded [10,11]. In this report, a three-year-old boy presented with RPE secondary to minor trauma to the soft palate, which is similar to a reported case by Wu K et al. in 2005 [12]. Dental procedures, especially when a high-speed handpiece with compressed air is used, are one of the reported causes of RPE as well as subcutaneous emphysema [9,13]. The literature also contains reports of retropharyngeal emphysema secondary to free-basing cocaine [14,15].

Facial fractures, including orbital, zygomaticomaxillary, antral, and mandibular fractures associated with pneumomediastinum have been historically reported in the literature, with or without retropharyngeal emphysema [16-19]. Surprisingly, nose blowing can be a precipitate cause of retropharyngeal and subcutaneous emphysema as reported by Dunn C in 2003 [20].

Since the case reported here was in a pediatric patient, the chief complaint is only elicited as pain and refusal to be fed, which is most likely attributed to odynophagia. However, clinical features of retropharyngeal emphysema are chiefly neck pain, sore throat, and odynophagia, as per the literature [10-12]. Chest pain, when present, may point to the presence of pneumothorax and/or pneumomediastinum [19]. Facial swelling can also be seen in the case of subcutaneous emphysema [20]. Comprehensive history taking and meticulous head and neck examination are of the utmost importance when suspecting retropharyngeal emphysema, a history of facial trauma, and evidence of local trauma to the palate and/or pharynx might be noted.

In this report, a linear laceration to the soft palate and posterior pharyngeal wall was evident, the Wu study reports the same physical findings [12]. A high index of suspicion is critical when a patient presents with a history suggestive of RPE. A lateral neck radiograph is invaluable for the diagnosis of retropharyngeal emphysema, air streaks, and lucency in the retropharyngeal space anterior to the cervical vertebrae and along the fascial planes [21]. When needed, computed tomography is helpful to further investigate emphysema and define its extension [22].

The management of RPE varies according to the complexity of the case; conservative management is usually sufficient unless complications develop. Isolated retropharyngeal emphysema, such as the case presented here, is usually self-limited and follows a benign course. Conservative treatment, including observation, analgesia, and broad-spectrum antibiotics, is the mainstay in case of uncomplicated RPE; supplemental oxygen is given only if indicated [12]. Surgical management is generally not recommended unless the patient shows no improvement in medical management or develops further complications such as severe pneumomediastinum and pneumothorax or infection [9,11]. A comparison of selected case reports found in the literature, including causes, associations, management, and outcomes, are summarized in Table 1.

**Table 1:** A comparison of selected retropharyngeal emphysema case reports found in the literature Abbreviations: Y: yes

Reference	Age (years)	Presumed etiology	Associations	Admitted	Specific management	Outcome
Our case	3	Soft palate and posterior pharyngeal wall injury	Nil	Y	Analgesia and antibiotics	Complete recovery
Brett Wu et al., 2006[10]	20	Spontaneous	Nil	Y	NPO and fluid hydration	Complete recovery
Wu et al., 2005[12]	23	Soft palate injury	Nil	Y	NPO, fluid hydration, and antibiotics	Complete recovery
Ong et al., 2005[17]	70	Left orbital and zygomaticomaxillary fractures	Cervicofacial and mediastinal emphysema	Y	Antibiotics and analgesia	Complete recovery
Riccio et al., 1990[15]	19	Free-basing cocaine	Nil	Y	Oxygen via a non-rebreather mask	Complete recovery
Mather et al., 2006 [9]	43	Dental procedure	Diffuse emphysema from infratemporal to mediastinal space	Y	Prophylactic antibiotics	Complete recovery
Dunn et al., 2003[20]	20	Nose blowing	Suspected orbital fracture and intra-orbital emphysema	Y	Decongestant and antibiotics	Complete recovery
Minton et al., 1984[19]	32	Mandibular fracture	Pneumomediastinum and pneumothorax	Y	Oxygen via nasal cannula and antibiotics, surgical management of fracture 3 days after	Complete recovery

## Conclusions

Retropharyngeal emphysema, the abnormal presence of air in the retropharyngeal space, is rarely encountered in clinical settings. It can be either spontaneous (primary) or trauma-induced (secondary). Such patients can be managed conservatively, with close monitoring, as was evident in our case. Complete recovery without any further complications is reassuring in the majority of cases. Further studies and case reports are encouraged to aid in establishing clear guidelines when managing a case of retropharyngeal emphysema.
